# Supplementary material for: Bidirectionally promoting assembly order for ultrastiff and highly thermally conductive graphene fibres
Source: Nat Commun. 2024 Jan 9;15:409. doi: 10.1038/s41467-024-44692-7 (PMC10776572; doi:10.1038/s41467-024-44692-7)
Supplement: Supplementary file 1 — Supplementary Information [file 41467_2024_44692_MOESM1_ESM.pdf]

Bidirectionally promoting assembly order for ultrastiff and highly thermally conductive graphene fibres

Peng Li[1, †], Ziqiu Wang[1, †], Yuxiang Qi[1, †], Gangfeng Cai[1], Yingjie Zhao[2], Xin Ming[1], Zizhen Lin[3], Weigang Ma[3], Jiahao Lin[1], Hang Li[1], Kai Shen[1], Yingjun Liu[1,4], Zhen Xu*[1,4], Zhiping Xu*[2], Chao Gao*[1,4]*

[1] MOE Key Laboratory of Macromolecular Synthesis and Functionalization, International Research Center for X Polymers, Department of Polymer Science and Engineering, Zhejiang University, 38 Zheda Road, Hangzhou 310027, P. R. China

[2] Applied Mechanics Laboratory, Department of Engineering Mechanics and Center for Nano and Micro Mechanics, Tsinghua University, Beijing 100084, P. R. China

[3] Key Laboratory for Thermal Science and Power Engineering of Ministry of Education, Department of Engineering Mechanics, Tsinghua University, Beijing 100084, P. R. China

[4] Shanxi-Zheda Institute of Advanced Materials and Chemical Engineering, Taiyuan 030032, P. R. China

***Corresponding authors:**

yingjunliu@zju.edu.cn (Y. L.);

zhenxu@zju.edu.cn (Z. X.);

xuzp@tsinghua.edu.cn (Z. P. X.)

chaogao@zju.edu.cn (C.G.)

Supplementary Text.

Supplementary Figures 1-27.

Supplementary Table 1-4.

Supplementary References.

Supplementary Text

1.1 Calculation of order parameter of graphene fibres.

The alignment of graphitic crystallites is quantified by converting the orientation distribution into Herman's orientation function, *f*, defined as[1]:

$$f =< \frac{3}{2} cos^2\varphi - \frac{1}{2} > \tag{1}$$

where, φ is azimuthal distribution of the (002) peak in WAXS pattens, and $cos^2\varphi$ is the average value of the square of the cosine of angle φ. Assuming rotational symmetry of the fibre axis, the following equation is obtained.

$$cos^2\varphi = \frac{\int_0^{\frac{\pi}{2}} I(\varphi) cos^2\varphi sin\varphi d\varphi}{\int_0^{\frac{\pi}{2}} I(\varphi) sin\varphi d\varphi} \tag{2}$$

where, $I(\varphi)$ is the intensity at φ in the azimuthal scanning curve.

1.2 Measurement of thermal conductivity of graphene fibres and aerogel fibres.

The thermal conductivities of graphene fibres and macroscopic graphene structures are measured by an optimized steady-state electrical heating method in a vacuum chamber[2-4]. In contrast to most studies where two electrodes are used, we here suspended a single fibre between four copper blocks which simultaneously served as heating sinks and electrodes. A Keithley 2611B source meter is used to supply direct current and to measure the voltage drop across the middle two electrodes by a standard four-wire method. This modified steady-state electrical heating method eliminates contact resistance, enabling much more reliability of testing results. The sample chamber is continuously evacuated by a molecular pump to maintain a high vacuum environment (~10^4 Pa) in order to eliminate convective heat transfer. When a direct current fixed on the suspended fibre, temperature will increase due to the Joule heating effect and be controlled lower than 60 °C. An infrared camera (FLIR T630sc) with a close-up lens is used to measure the temperature profile along with the fibre through a zinc selenide viewport (Supplementary Fig. 27). Hence, the thermal conductivity of the measured fibre is calculated as:

$$\kappa = \frac{UIL}{4A_c(T_0 - T_a)} \tag{3}$$

where, κ is the thermal conductivity, U and I are the voltage and current measured across the middle two copper electrodes, L is the half-length between the middle two contacts. A_c is the cross-

section area of the fibre. T_o is the temperature at the middle point of the measured fibre and T_a is that at an edge point.

The thermal conductivity of graphene fibres was further examined by a T-type method, which is suitable for thin fibres with diameter of 5-7 μm[5]. A Platinum hot wire suspended on two heat sinks was served as temperature sensor and one end of GF was attached to the centre of the hot wire, while the other end was linked to a heat sink. The sample chamber was continuously evacuated by a molecular pump to maintain a high vacuum level of 10^{-4} Pa. The temperature of the sample holder was steadily controlled by temperature controller (Oxford Instrument, ITC503) with an accuracy of ±0.1 K. The measurement system consists of the sample, a standard resistance, a high accuracy constant power supply (Advantest R6243), and two high accuracy digital meters (Keithley 2002). The constant power supply provides direct current and the digital meters were used to detect the voltages on sample and standard resistance. When the DC current is imposed on the hot wire without the fibre, a parabolic temperature distribution is built. After attaching the graphene fibre to the hot wire, heat was partly transported from the hot wire to the fibre and the temperature distribution became dual-arch. Comparing the average temperature change of hot wire before and after the attachment of graphene fibre, the thermal conductivity can be extracted,

$$\lambda_f = \frac{\lambda_h A_h l_h l_f (q_v l_h^3 - 12\lambda_h l_h \Delta T_v)}{A_f l_{h,1} l_{h,2} [12\lambda_h l_h \Delta T - q_v (l_{h.1}^3 + l_{h,2}^3)]} \quad (4)$$

where λ_h and λ_f are the thermal conductivities of the hot wire and the graphene fibre, respectively. A_h and l_h are the cross section and length of the hot wire, respectively. $l_{h,1}$ and $l_{h,2}$ are the lengths of the left and right part of the hot wire, respectively. A_f and l_f are the cross section and length of the graphene fibre, respectively. ΔT_v is the average temperature rise of the hot wire determined from the resistance change. $q_v = IV/(A_h l_h)$ is the volumetric heat generation rate of the electric current heating. I and V are the applied current and voltage, respectively. In the measurements, a platinum wire (99.99%, Alfa Aesar) with diameter of 25 μm is used as hot wire. Before adhering the test sample, the physical properties of platinum hot wire were calibrated applying the DC heating method. The temperature coefficient of resistance and thermal conductivity of hot wire are measured in advance by $\beta_T = \Delta R/(R_0 \Delta T)$ and $\lambda = I_h^2 q_v/(12\Delta T_v)$, respectively. The measured average temperature coefficient of resistance of platinum hot wire is 0.0037 K^{-1} in

the range of 273-298 K and the thermal conductivity of platinum hot wire is 77.2 W m^{-1} K^{-1} at 298 K.

1.3 Calculation of microvoids in graphene fibres.

The parameters of microvoids were calculated by Ruland's streak method with Gaussian distribution function[6, 7].

$$s^2 B_{\pi/2}^2(s) = 1/L^2 + s^2 B_{eq}^2 \tag{5}$$

where, $B_{\pi/2}(s)$ is the integration breadth along azimuthal scan, s is the scattering vector $s = 2\sin\theta/\lambda$, B_{eq} is the misorientation angle representing the preferred orientation of the microvoids and L is the microvoid length.

The value of L and B_{eq} can be obtained from the intercept and slope of the $s^2 \sim s^2 B_{\pi/2}^2(s)$ plot defined in the above equation. With the approximation, we finally obtain

$$I(s, \pi/2) \propto n\rho_m^2 \frac{L^2}{\sqrt{1+\left(sLB_{eq}\right)^2}} |\Phi_D|^2(s) \tag{6}$$

where,

$$|\Phi_D|^2(s) \propto \frac{l_p^4}{\left[1+\left(2\pi l_p s\right)^2\right]^3} \tag{7}$$

Inserting the calculated values of L and B_{eq}, the number of microvoids, n, and the average chord length in the cross-section of microvoids or diameter for short, l_p, can be calculated. Then the total volume of microvoids V can be approximately be evaluated by $V \propto nLl_p^2$.

1.4 Simulation of sheet arrangement in the multiple shear field.

We performed a computational fluid dynamics (CFD) simulation to analyse the effect of rotating angular velocity on graphene sheet arrangement in the multiple shear-flow field [8]. The geometry parameters and the density/dynamic viscosity of graphene oxide solution were considered according to experimental condition. Shear stress transport (SST) k-omega turbulence model belonging to the family of Reynolds-averaged Navier-Stokes (RANS) two-equation model is adopted to depict the effect of turbulent flow conditions [9]. The coupled scheme is utilized to address the pressure-velocity coupling for the discretized form of the Navier-Stokes system, and the Green-Gauss cell based method is used for gradients estimation [10].

1.5 Simulation of fibres formation.

We conducted a two-dimensional (2D) coarse-grained molecular dynamics (CGMD) simulation, using the stacking patterns of graphene chains to represent the cross-sectional shrinkage.

Subdomains containing axial and radial atoms in graphene are gathered into beads with the equal mass. Adjacent beads are connected by bonds, and the balanced distance r_{b} is set to 2 Å. The balanced angle α between adjacent bonds is set to 180°. Utilizing the bond energy in the model to be equal to its corresponding continuous medium stretch energy, we obtained the tensile stiffness $k_{\mathrm{s}}=\frac{1}{2}\frac{l}{r_{\mathrm{b}}}Yt$; according to the angle energy in the model to be equal to its corresponding continuum bending energy, we determined the bending stiffness $k_{\mathrm{b}}=\frac{1}{2}\frac{l}{2r_{b}}D$. Among them, Y and D are the Young's modulus and bending stiffness of graphene oxide, respectively; t is the thickness of graphene, and l is the length related to the graphene size L. We set $l=0.05L$, and assume that the strain and curvature do not vary with the change of the axial position as the axial size in the range of l. Y is set to 200 GPa and D is set to 2 $k_{\mathrm{B}}T$ since graphene oxide are quite flexible because of functional groups [11]. t is set to 4 Å and L is set to around 10 nm. For the interaction between non-bonded beads, we used the Lennard-Jones 12-6 potential function, $U=4\varepsilon[(\sigma/r)^{12}-(\sigma/r)^{6}]$, where the parameters σ and ε are determined by fitting the balanced inter-layer spacing and cohesive energy, respectively. The simulated parameters are shown in Supplementary Table 1. The simulation is performed utilizing the large-scale atomic/molecular massively parallel simulator (LAMMPS) [12]. To simulate the formation process of fibres, we compress the graphene chains with circular constraint at 300 K until the layer spacing reaches equilibrium (circular section). The Langevin thermostat was used to control the temperature.

1.6 Crystalline model of graphene fibres.

TEM images of the axial direction reveal bundles of crystalline domain in the fibre and single crystallites in a graphitic bundle (Supplementary Fig. 14b-d). We then proposed a crystalline model and key structural factors of graphene fibre as schemed in Supplementary Fig. 14a. Generally, graphene fibre consists of giant graphene sheets with a macro-structural factor of density (ρ); these giant graphene sheets pile into continuous graphitic bundles with misoriented angle (α) and calculated orientation degree (f); perfectly stacked sheets in a bundle form single graphitic crystallites with three-dimensional crystalline size, including thickness (Lc), axial length ($La_{/\!/}$), and transverse length ($La_{\perp}$). Based on the proposed crystalline model and crystalline structural factors, we analysed the crystallinity of the fabricated graphene fibres systematically. Optimized concentric texture at ω=100 (×2π/60) rad/s is in favour to form ordered sheet-arrangement in both axial and

transverse direction in final shrinked graphene fibre, which results in an improved order parameter (0.93) and enlarged crystallite sizes (Lc=68.5 nm, $La_{/\!/}$=236.6 nm, $La_{\perp}$=114.1 nm). The MSW strategy improves the sheet-arrangement order in the fibre cross-section compared to previously reported graphene fibres, thus distinctly facilitates the growth of graphitic crystallites along the thickness (Lc) and transverse length ($La_{\perp}$). Concentric graphene fibre prepared at ω=100 (×2π/60) rad/s has increase rate of Lc, $La_{\perp}$, and $La_{/\!/}$ reaching 235%, 74%, and 31% compared to these without MSW spinning, respectively (Supplementary Fig. 16d).

Supplementary Figures

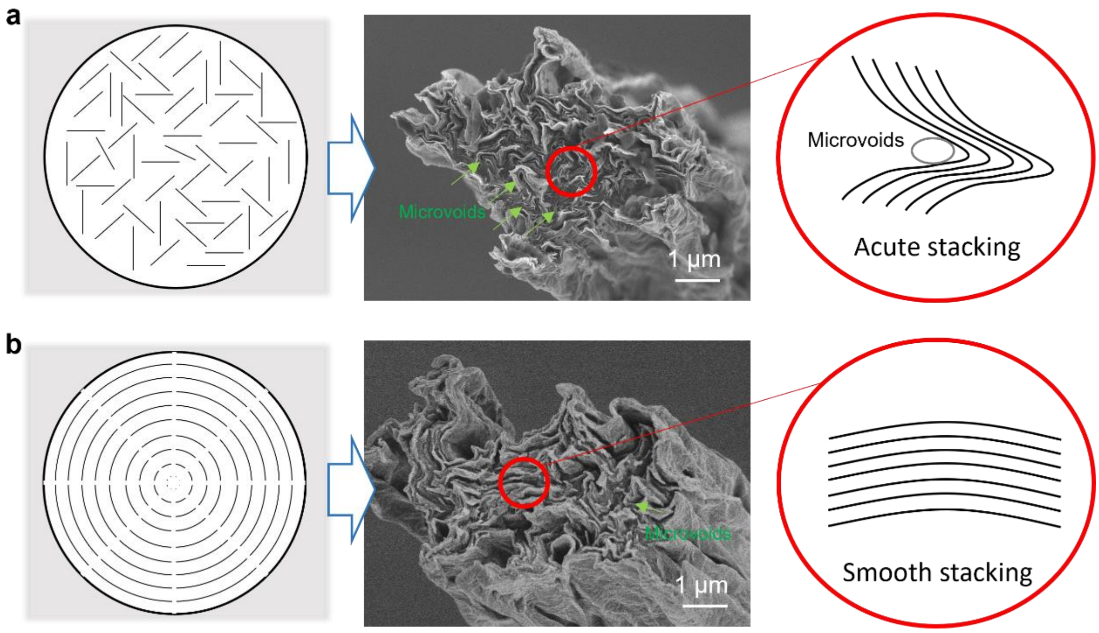

Supplementary Figure 1. Distinction in graphene fibres fabricated from random and concentric texture. a-b, Schematics and SEM images of graphene fibre coagulated and solidified from random (**a**) and concentric (**b**) GO gel fibre, depicting the initial motivation. Graphene fibre from random sheet-ordered GO structure shows distinctly and widely distributed acute stacking, making graphene sheets loosely stacked and resulting in microvoids mainly at the centre of acute stacking, while graphene fibre from concentric sheet-arranged GO structure shows smoother sheet-stack, managing graphene sheets optimally to higher density. Smooth stacking of graphene sheets also facilitates the improved graphitization with enlarged three-dimensional crystalline sizes.

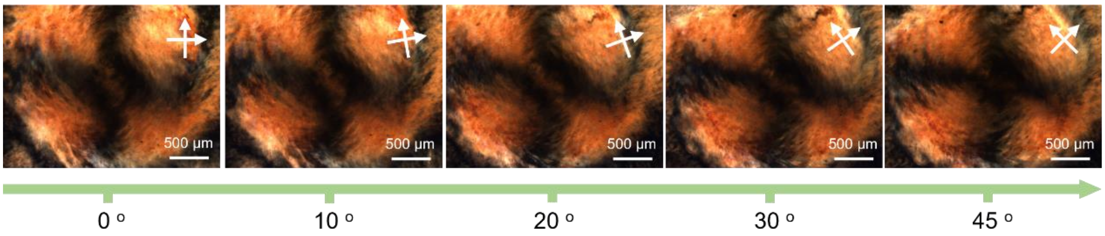

Supplementary Figure 2. POM images of GO liquid crystal at rotating angular velocity of 100 (×2π/60) rad s^{-1} at polarizing angle from 0° to 45°, showing typically concentric roll texture.

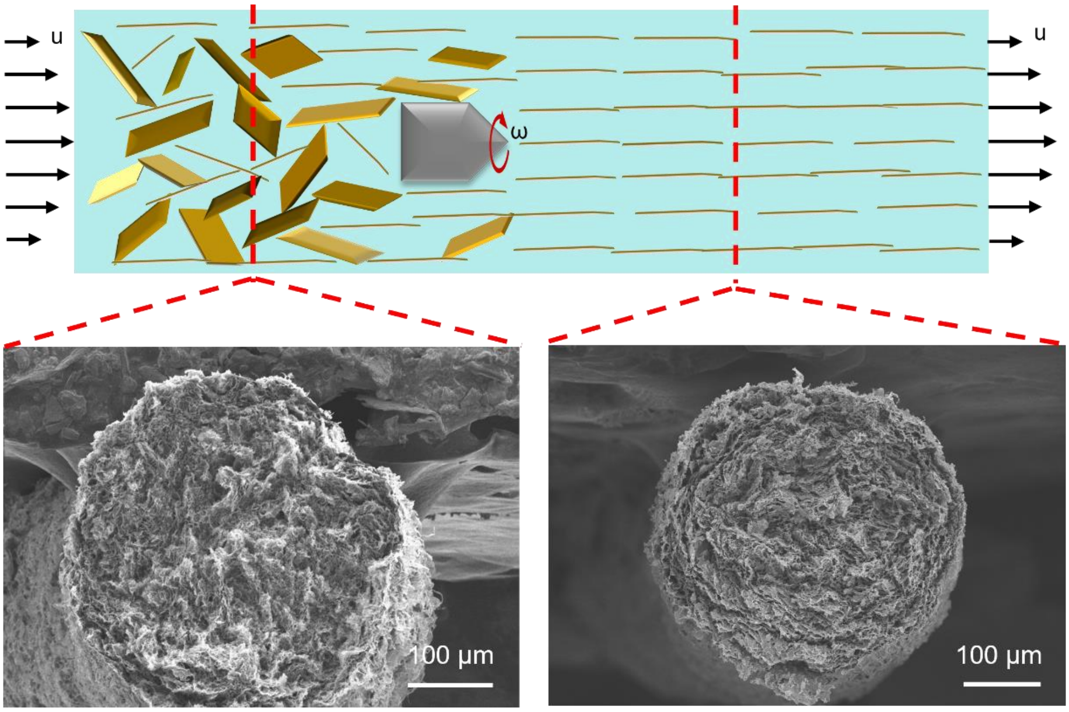

Supplementary Figure 3. Structural tracking of the transformation of GO sheet-order in the multiple shear-flow fields.

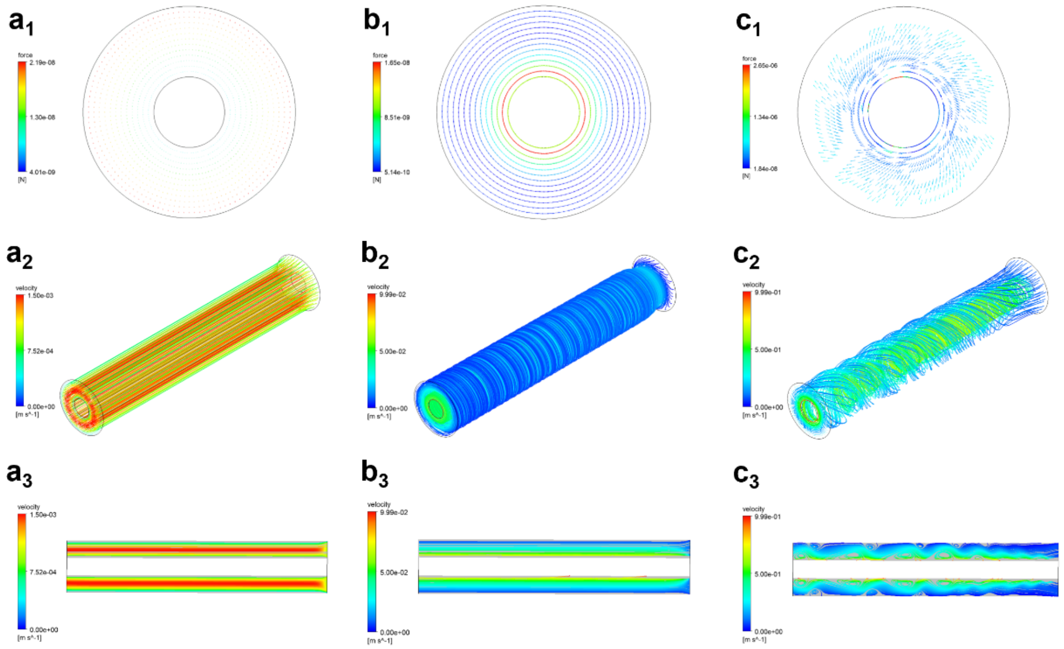

Supplementary Figure 4. Shear-flow distribution. $\mathbf{a_1}$-$\mathbf{c_1}$, Shear force distribution of unidirectional tubular flow ($\mathbf{a_1}$) and multiple shear-flow with rotating angular velocity of 100 rad s^{-1} ($\mathbf{b_1}$) and 1000 rad s^{-1} ($\mathbf{c_1}$). $\mathbf{a_2}$-$\mathbf{c_2}$, Velocity distribution of unidirectional tubular flow ($\mathbf{a_2}$) and multiple shear-flow with rotating angular velocity of 100 rad s^{-1} ($\mathbf{b_2}$) and 1000 rad s^{-1} ($\mathbf{c_2}$). $\mathbf{a_3}$-$\mathbf{c_3}$, 2D velocity distribution along the tube axis of unidirectional tubular flow ($\mathbf{a_3}$) and multiple shear-flow with rotating angular velocity of 100 rad s^{-1} ($\mathbf{b_3}$) and 1000 rad s^{-1} ($\mathbf{c_3}$).

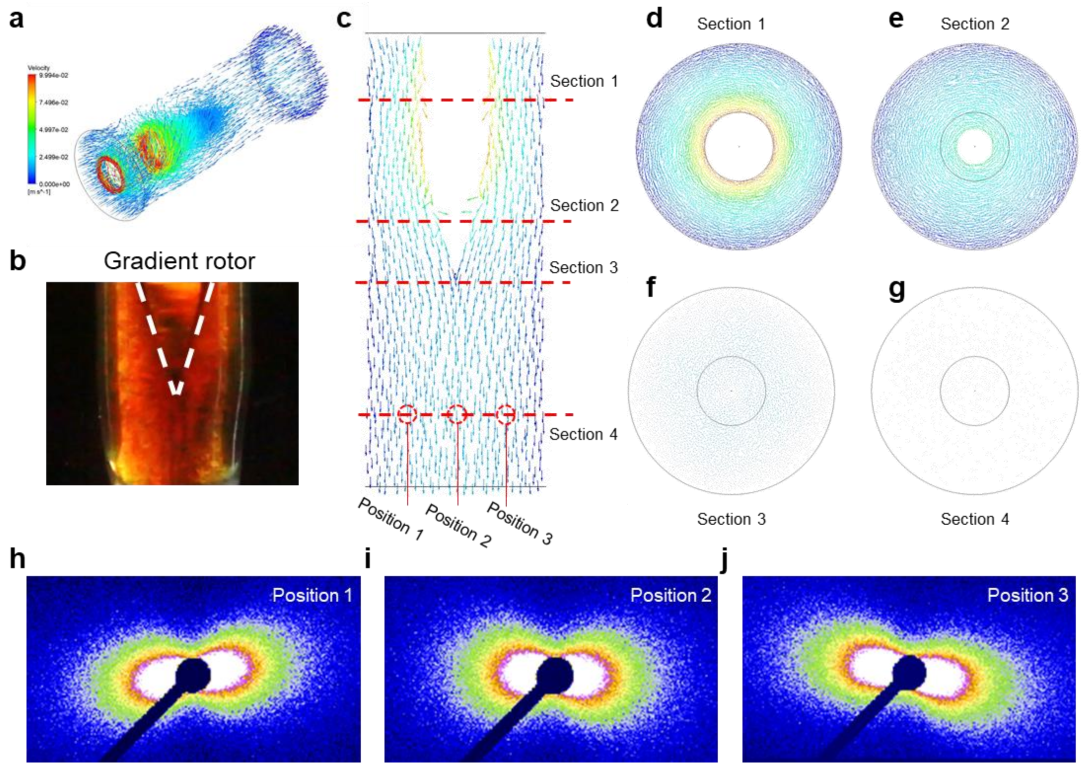

Supplementary Figure 5. The formation of concentric textured liquid crystals. a, c-g, Velocity distribution of multiple shear-flow fields with a gradient rotor. **b**, Observation of liquid crystal in multiple shear-flow fields with the gradient rotor. **h-j**, Position-resolution SAXS patterns showing the highly aligned GO sheets at both outer region (**h** and **j**) and core region (**i**).

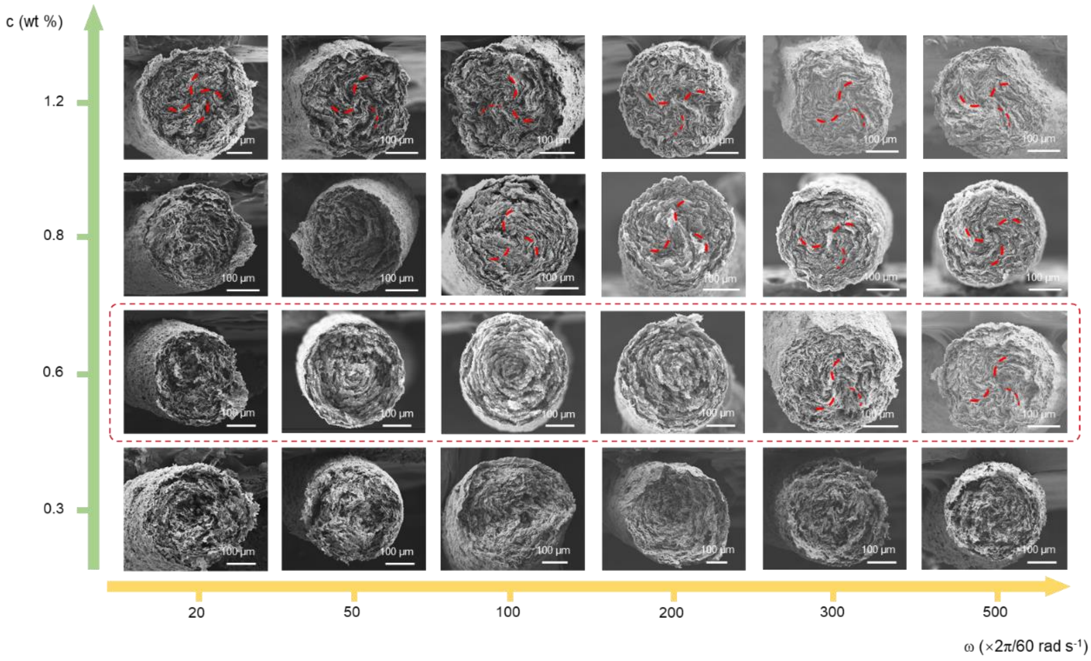

Supplementary Figure 6. Macroscopic graphene structure with variable sheet-arranged cross-section manipulated by applied flow field and GO concentration.

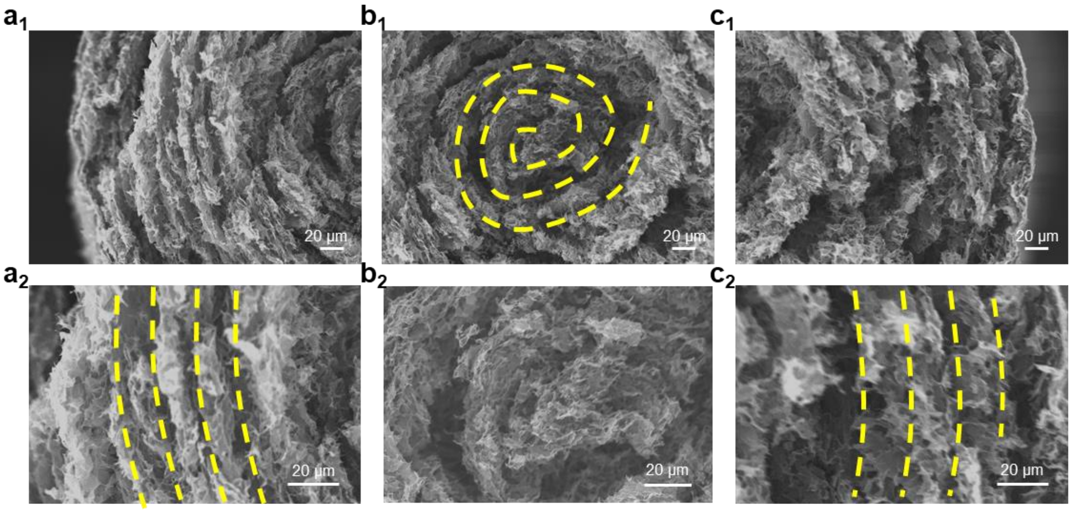

Supplementary Figure 7. SEM images of the concentric GO aerogel fibre. a_1-c_1, SEM images of different regions in concentric GO aerogel fibre. **a_2-c_2**, Corresponding zoomed-in images of core and outer regions. 2D sheets in the core region are bent with smaller curvature radius than those at the outer region, even away from an ideal concentric order, which form in the converging process in the tip of gradient rotor and mechanical disturbance of rotor. But the centre region is not empty or not loosely packed under a centrifugal force in the multiple shear-flow field.

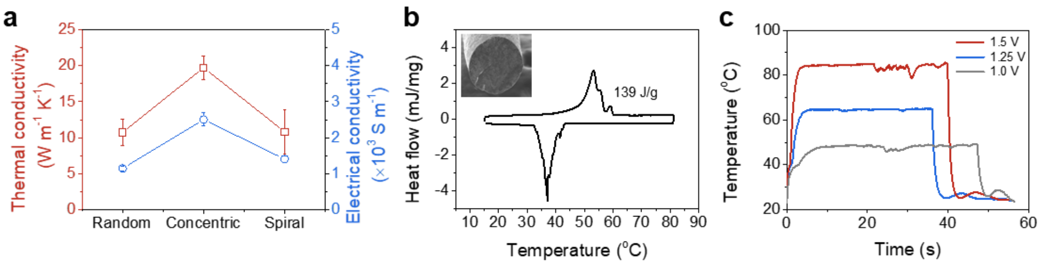

Supplementary Figure 8. Functional properties of macroscopic graphene structure after annealing at 2700 °C. a, Thermal and electrical properties of the graphene aerogel fibres featuring random, concentric, and spiral sheet-order. Error bars represent s. d. of the measured conductivities. **b**, Differential scanning calorimetry (DSC) file of the phase-change material composing the framework of concentric graphene aerogel fibre and PEG (94 wt%). **c**, Temperature increases of the phase-change fibre material at the external electron stimuli.

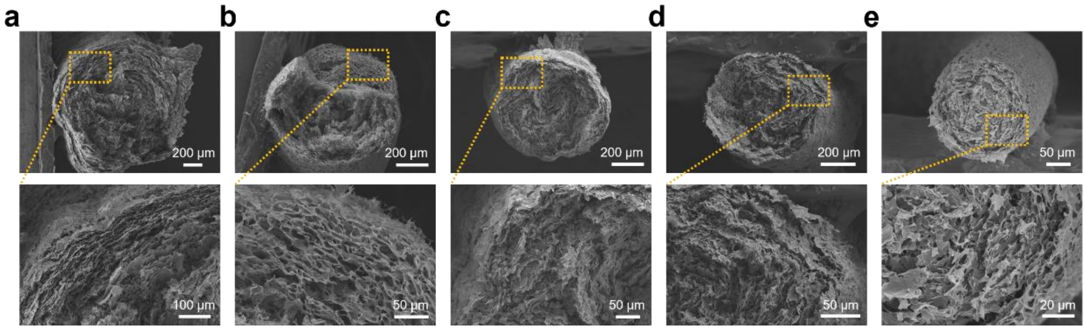

Supplementary Figure 9. Morphology of macroscopic graphene structures with variable diameter. a-e, SEM images of cross-sections of GO aerogel fibres prepared with different diameter of spinneret at the same rotating angular velocity.

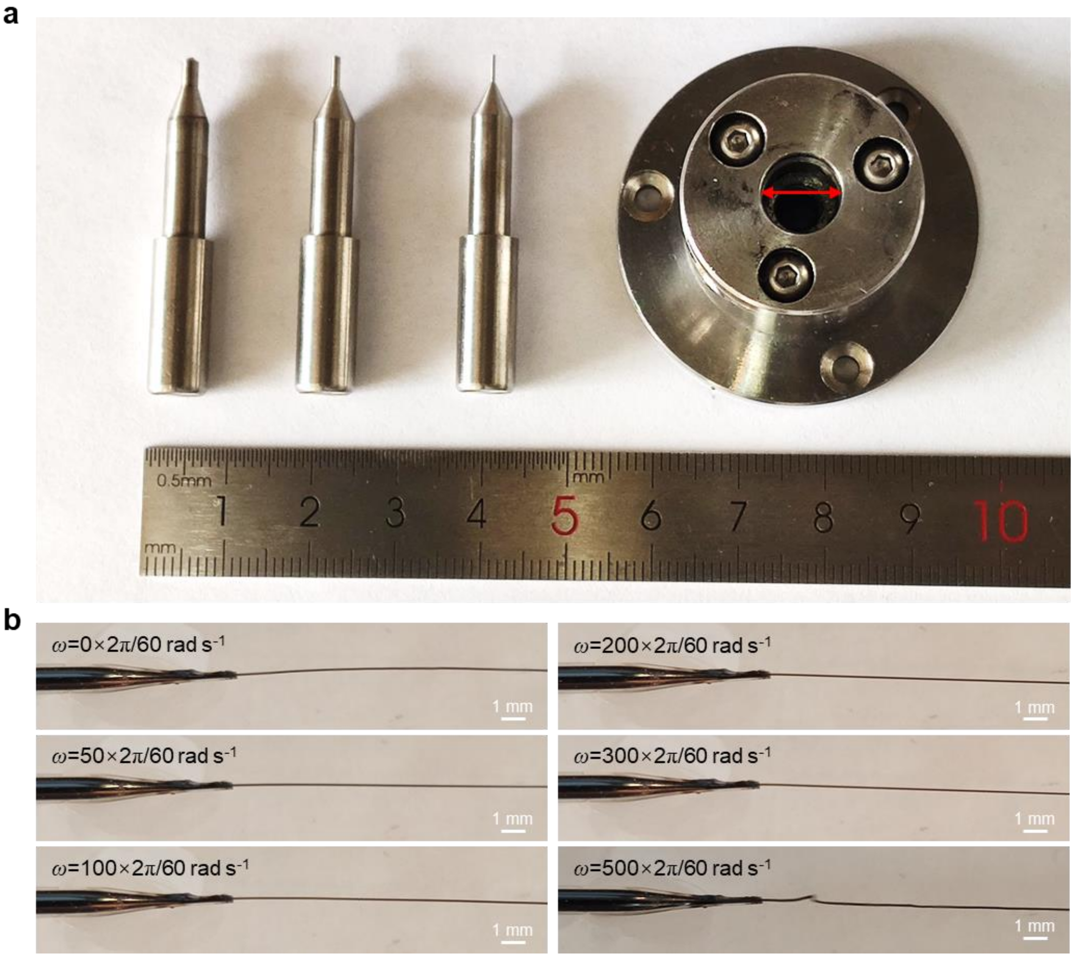

Supplementary Figure 10. MSW apparatus and its stable spinning process. a, Photograph of the home-made improved MSW apparatus combining the tubular and rotating shear fields. **b**, Stability of the fibre spinning process at externally introduced rotating angular velocity.

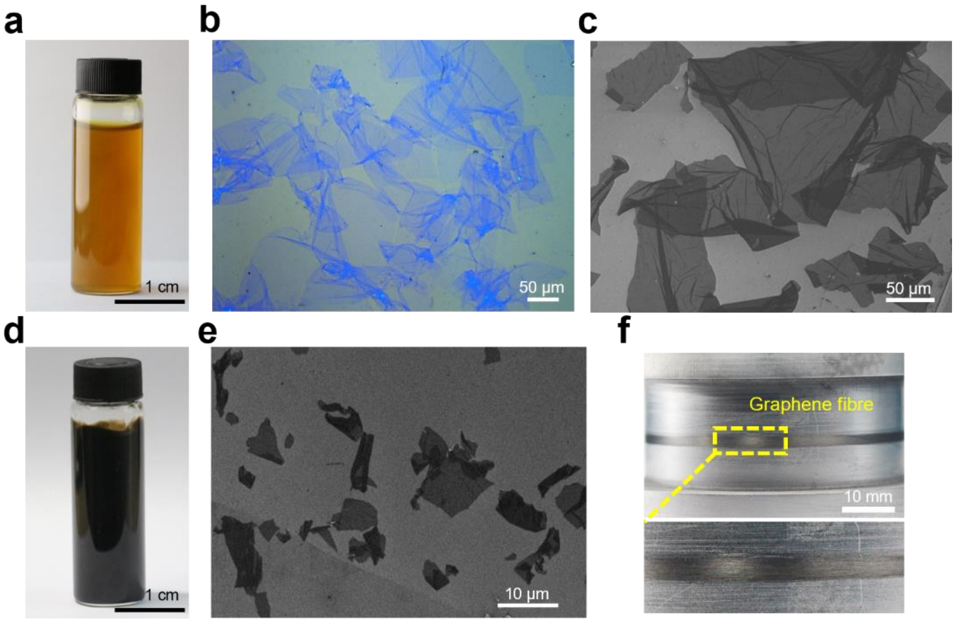

Supplementary Figure 11. Characterization of GO sheets and prepared graphene fibres. **a**, Photograph of large-sized GO solution. **b**-**c**, POM (**b**) and SEM (**c**) images of the large-sized GO sheets. **d**, Photograph of small-sized GO solution. **e**, SEM image of the small-sized GO sheets. **f**, Photograph of the prepared graphene fibre.

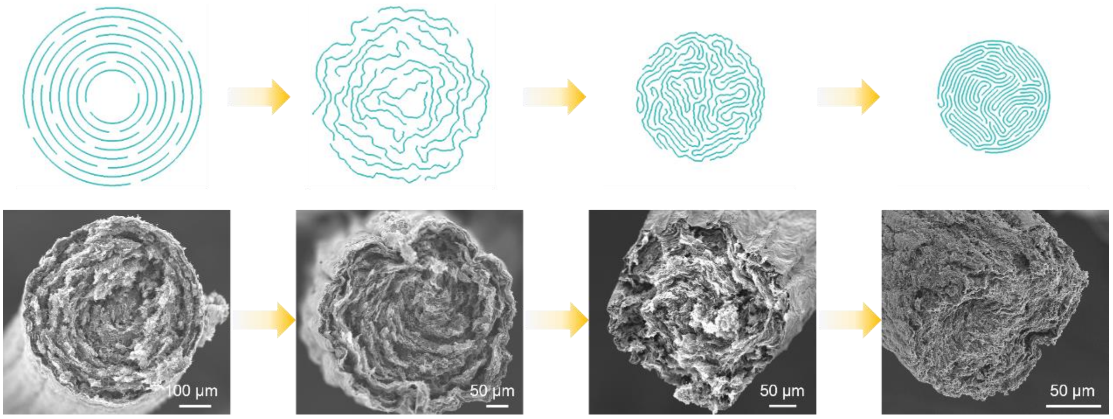

Supplementary Figure 12. 2D CGMD simulation (top) and SEM images (down) illustrating the shrinkage process in fibre solidification. In the solidification process of a GO fibre, large volume shrinkage of at least 100-fold (concentration of GO dope is 0.6 wt%) inevitably leads to an uneven folding under severe compressive capillary forces. Folded sheets form in both outer and centre regions with local small curvature radius. Finally, folded sheets stack together to a dense cross-section without clear central pore.

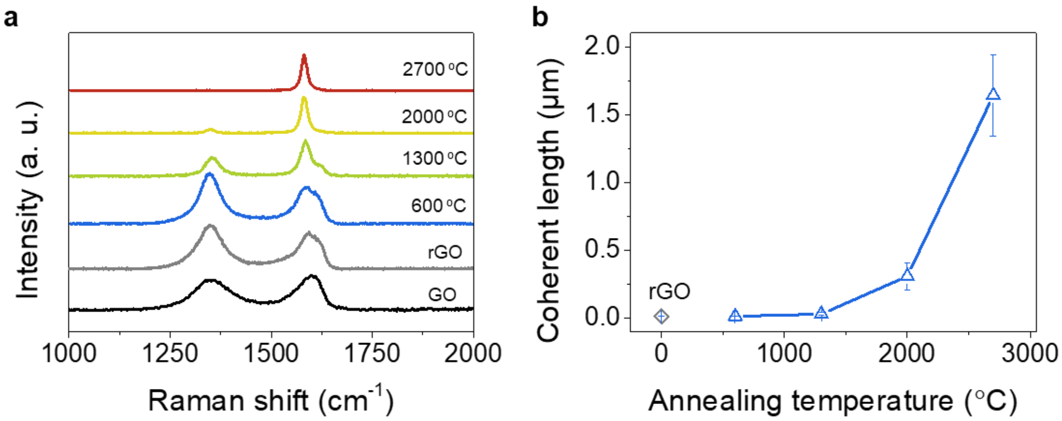

Supplementary Figure 13. Raman results depicting the restoration of defects. a, Raman spectroscopy of GO fibre, chemically reduced GO (rGO) fibre, and graphene fibres annealing at different temperature. **b**, Calculated coherent crystalline length from the measured Raman spectroscopy. Error bars represent s. d. of the calculated coherent length of concentric graphene fibre annealing at different temperatures.

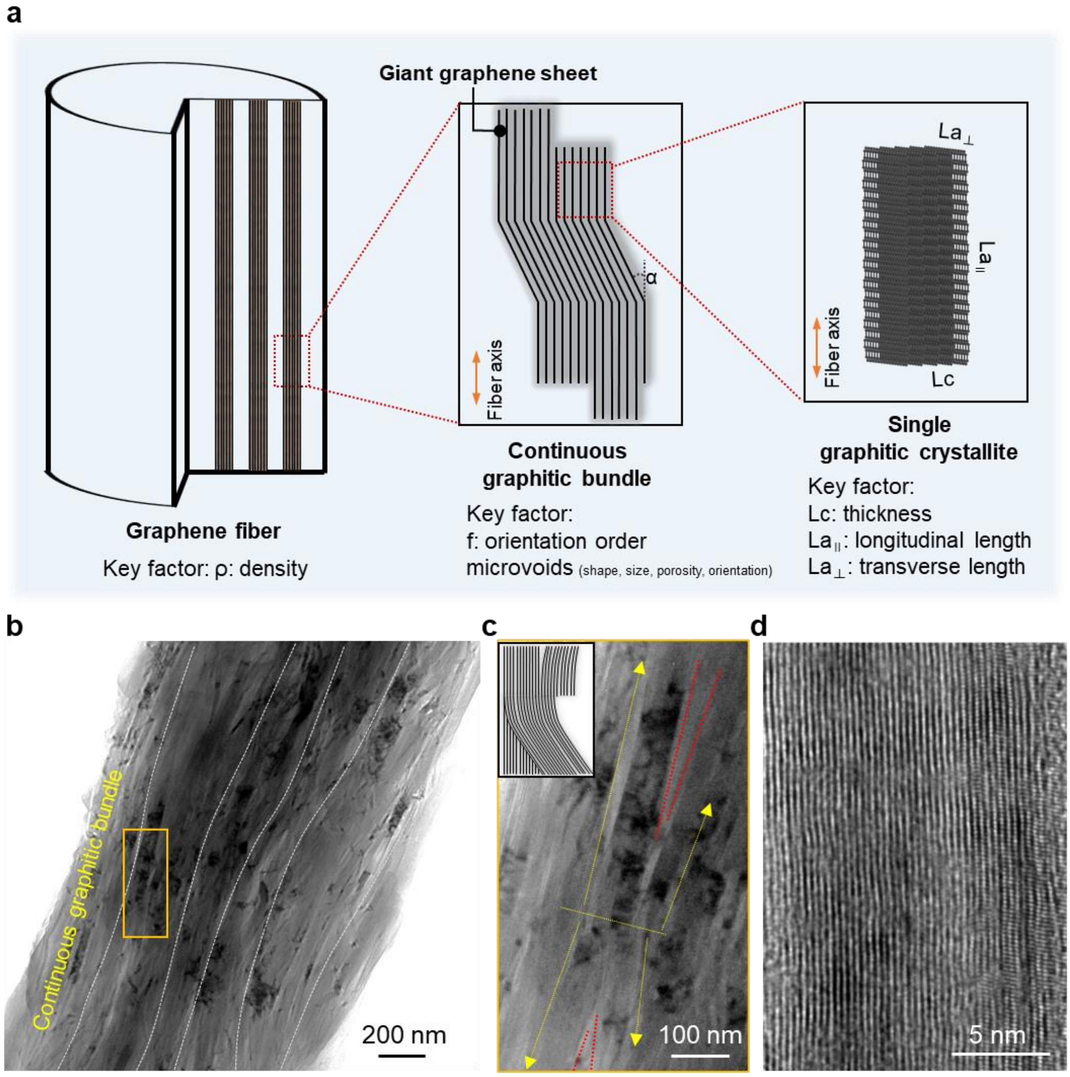

Supplementary Figure 14. Crystalline analysis of the graphene fibre. a, Crystalline model of the graphene fibre. **b-d**, Multi-scale TEM images of graphene fibre. Insert of (**c**) is a schematic illustration of continuous graphitic bundles with misoriented grain boundaries. The structure observed in TEM images verifies the proposed multi-scale crystalline model of graphene fibre.

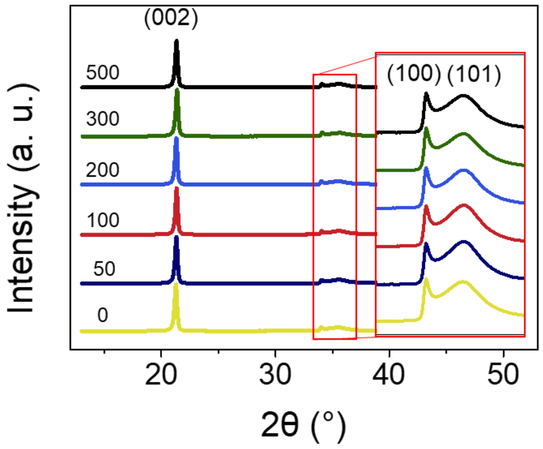

Supplementary Figure 15. The intensity of wide-angle X-ray scattering as a function of scattering angle (2θ) for an incident X-ray (wavelength λ of 0.124 nm) to bundles of graphene fibres prepared at different rotating angular velocity ω.

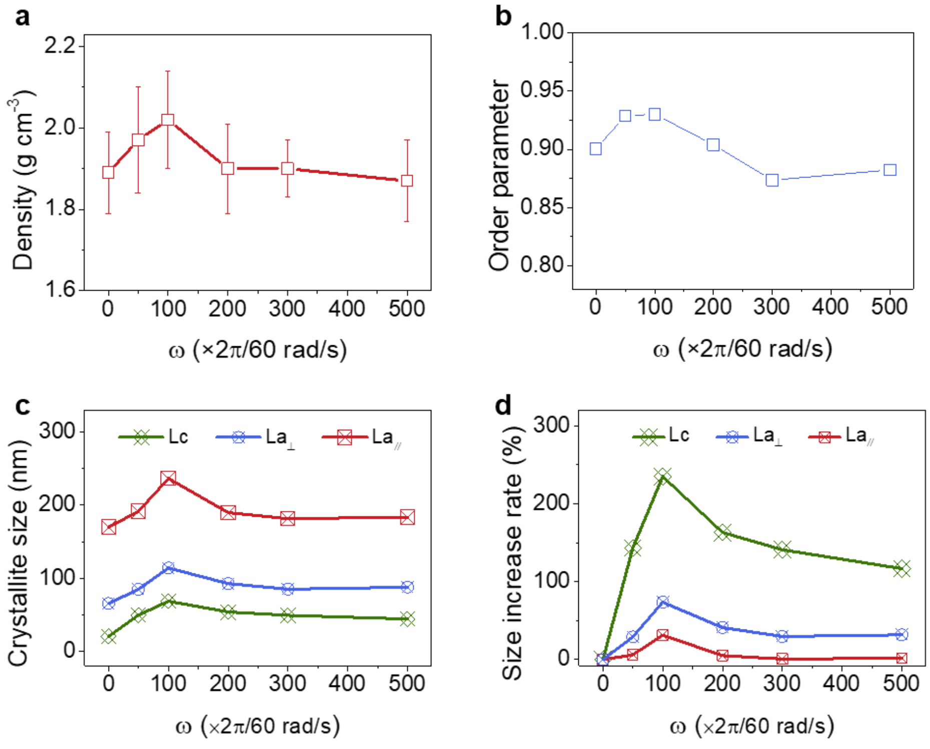

Supplementary Figure 16. Characterization of the graphene fibre prepared with variable rotating angular velocity, including density (**a**), order parameter (**b**), crystalline size (**c**), and size increase rate (**d**). Error bars in **a** represent s. d. of the tested density.

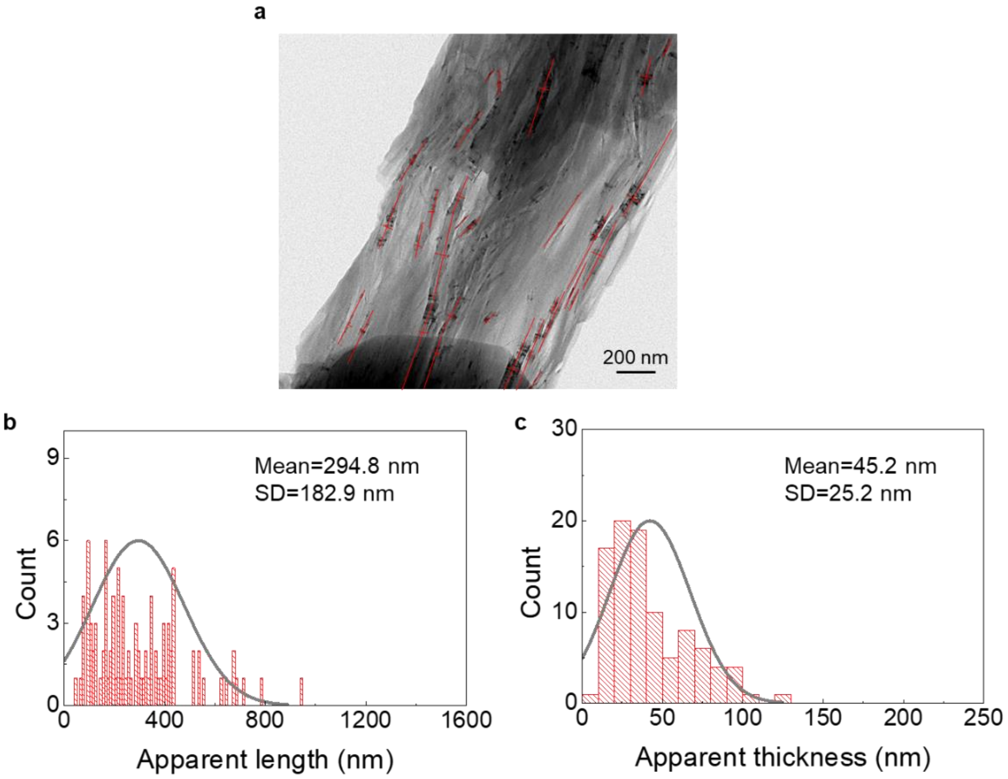

Supplementary Figure 17. Crystallites size of the concentric graphene fibre collected from TEM images. a, TEM image of the graphene fibre. **b-c**, Statistically apparent length and thickness of the graphene fibre.

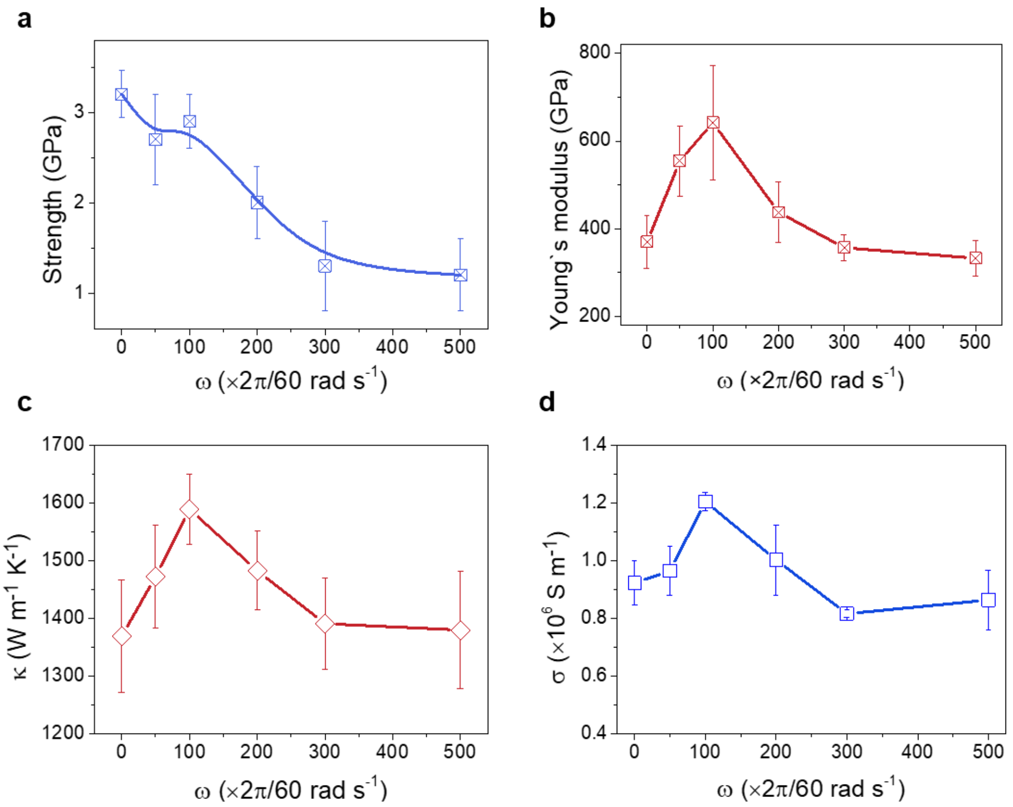

Supplementary Figure 18. Properties of the graphene fibre prepared with variable rotating angular velocity, including tensile strength (**a**), Young's modulus (**b**), thermal conductivity (**c**), and electrical conductivity (**d**). Error bars represent s. d. of the tested properties.

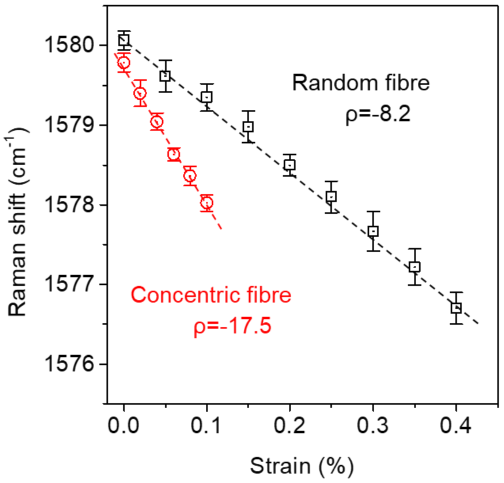

Supplementary Figure 19. In-situ Raman shift of G-band of concentric and random graphene fibres under tension. Error bars represent s. d. of the measured G-band position.

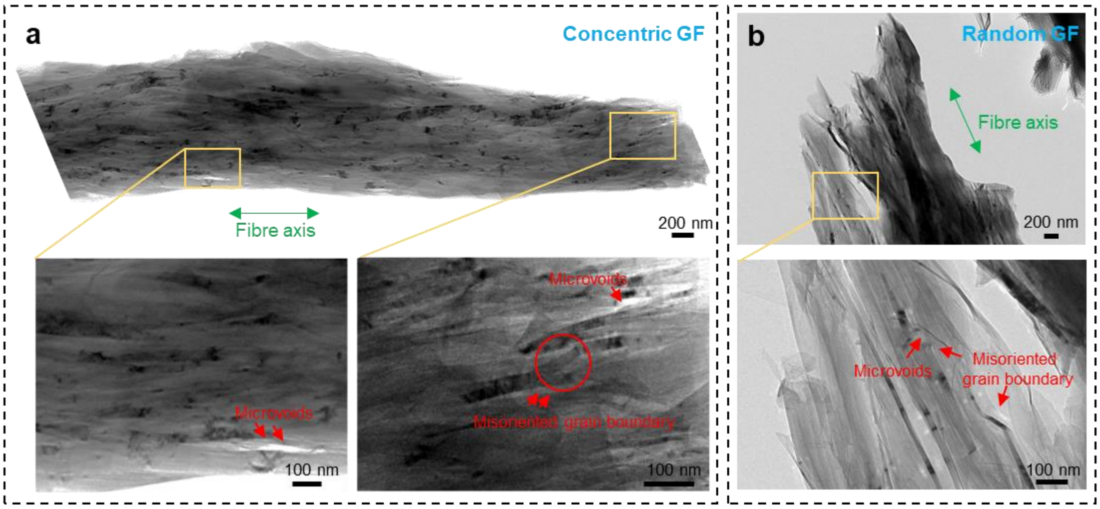

Supplementary Figure 20. TEM images of the axial section in concentric graphene fibres (**a**) and random graphene fibres (**b**) illustrating the main defects inside the fibres.

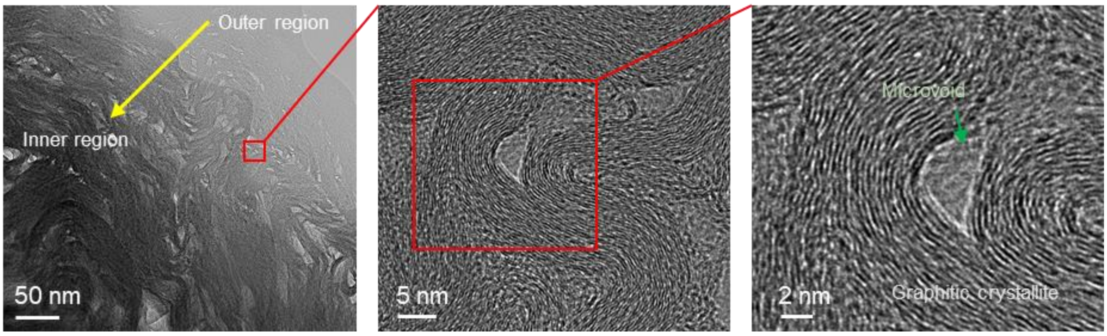

Supplementary Figure 21. TEM images of the transverse section in graphene fibres.

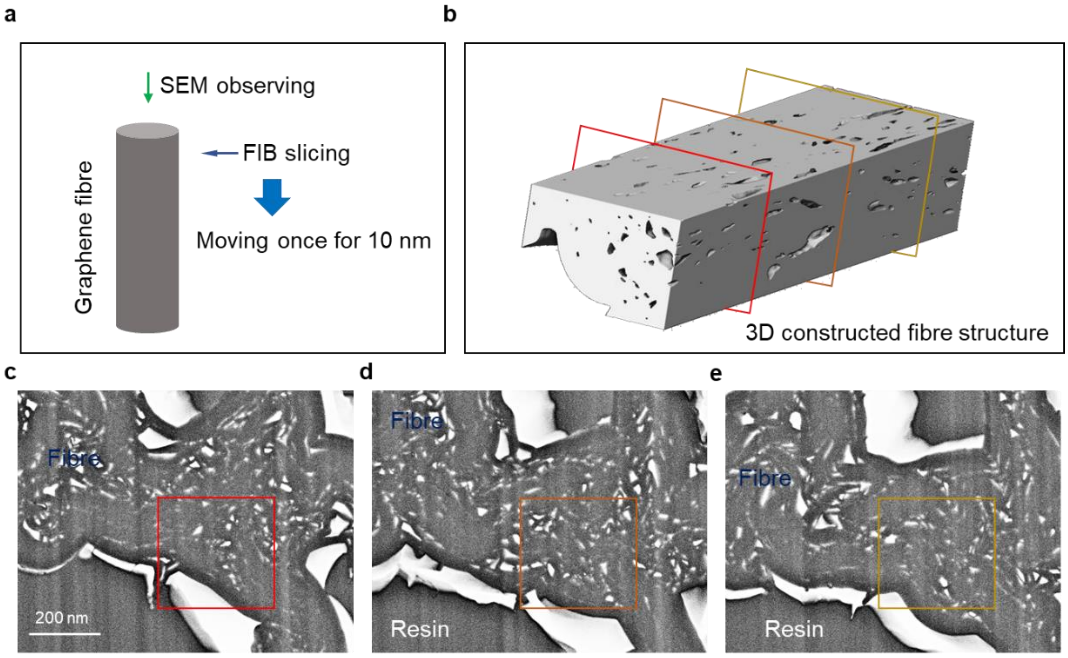

Supplementary Figure 22. 3D construction of concentric graphene fibre. a, Schematic depicting the 3D construction process. **b**, 3D constructed image of partial graphene fibre. **c**-**e**, SEM images of transverse cross-section representing the red, orange, and yellow boxes in **b**.

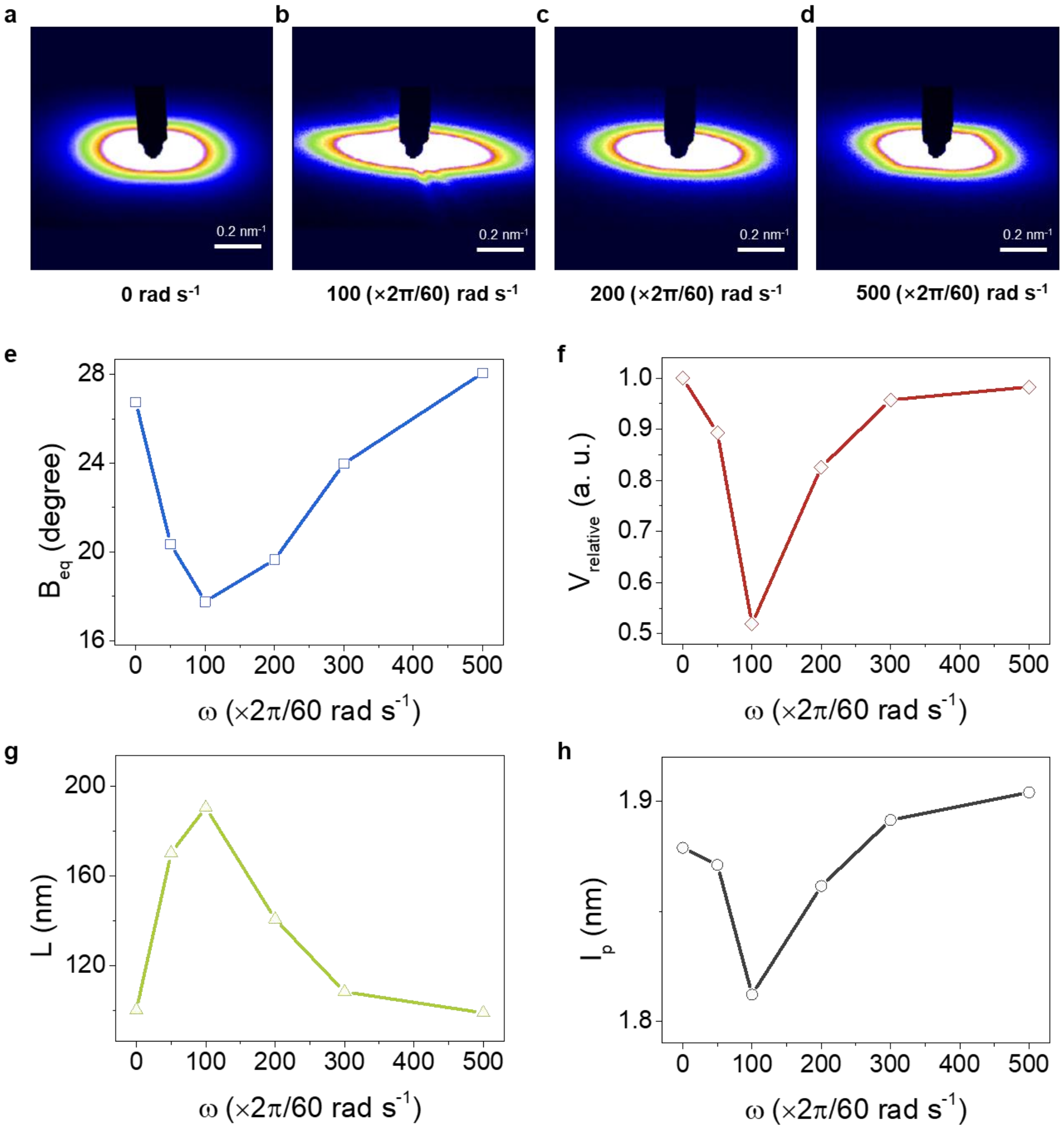

Supplementary Figure 23. Analysis of microviods in graphene fibres. a-d, SAXS patterns of graphene fibres fabricated at different rotating angular velocity. **e-h**, average misoriented angle (**e**), relative volume (**f**), length (**g**), and average chord length (**h**) of microvoids inside graphene fibres.

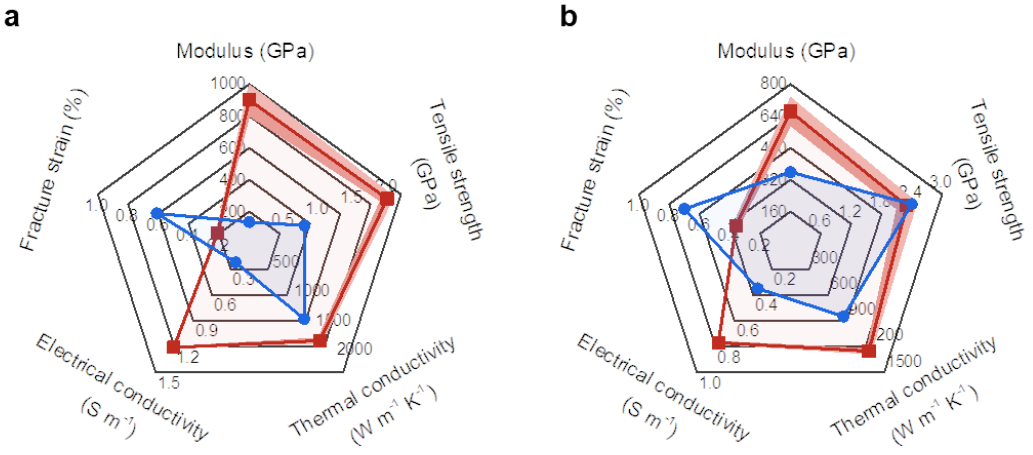

Supplementary Figure 24. The mechano-thermal properties of concentric carbonaceous fibres. a, Properties of concentric graphene fibre (red point plots) and reported random graphene fibre[2] (blue point plots) with the same 70 wt% large-sized GO and 30 wt% small-sized GO. **b**, Properties of concentric PAN/GO carbon fibre (red point plots) and reported random carbon fibre[4] (blue point plots) with the same 70 wt% PAN chains and 30 wt% GO sheets. Error bars in **a** and **b** represent s. d. of the measured properties.

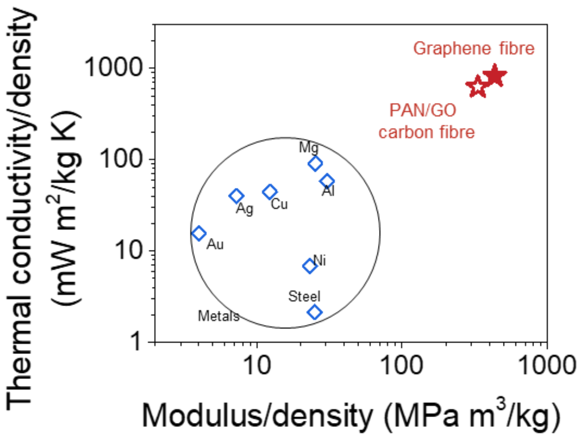

Supplementary Figure 25. Specific thermal conductivity and modulus of the concentric graphene fibres and PAN/GO carbon fibre, comparing with the conventional metals.

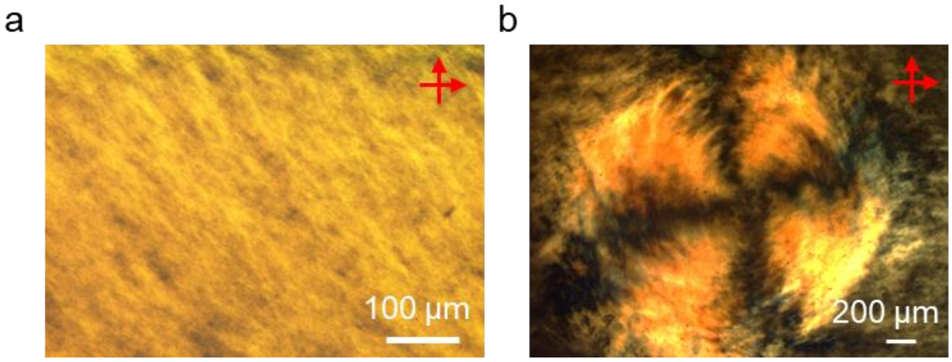

Supplementary Figure 26. POM images of the PAN/GO spinning dope with 70 wt% PAN chains and 30 wt% GO sheets. a, POM image of liquid crystalline PAN/GO solution. b, POM image of the concentric roll texture of the PAN/GO liquid crystals.

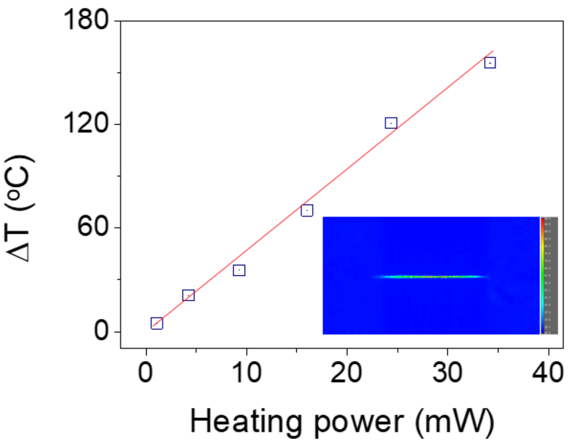

Supplementary Figure 27. Thermal conductivity measurements. The change of temperature difference between the middle of the fibre and the end of the fibre with respect to input power, insert image is the thermal infrared image of the self-heated graphene fibre.

Supplementary Table 1. Parameters of 2D CGMD simulation.

Parameters	Units	Values
Balanced inter-bead distance, r_b	Å	2
Tension stiffness, k_s	$kcal/(mol \cdot Å^2)$	287
Balanced angle, α	degree	180
Bending stiffness, k_b	kcal/mol	1.5
Lennard-Jones 12-6 function parameters, σ	Å	3.67
Lennard-Jones 12-6 function parameters, ε	kcal/mol	3.94

Supplementary Table 2. Crystalline parameters, density, modulus, and thermal conductivity of graphene fibres prepared by bidirectionally promoting assembly order, previously reported graphene fibres, and conventional carbon fibres involving polyacrylonitrile (PAN)-based carbon fibre and Phase-based carbon fibre.

Category	Lable	$La_{//}$ (nm)	Density (g cm^{-3})	Modulus (GPa)	Thermal conductivity (W m^{-1} K^{-1})	Reference
Graphene fibres	Random	170.2	1.89	370	1369	This work
	Concentric	236.6	2.02	642	1590	
	Spiral	183.8	1.87	333	1380	
	32%SR	174.3	1.89	341	1480	[13]
	27%SR	118.0	1.82	243	1280	
	14%SR	76.5	1.6	179	1140	
	0%SR	53.8	1.45	110	800	
	GF-2850	783[a]	1.74	135	1290	[2]
PAN-based carbon fibres	T300	2.98	1.76	230	10.5	[4]
	T700	3.14	1.8	230	9.4	
	T1000	3.16	1.8	294	32	
	M30J	-	1.73	294	31.9	
	M35J	-	1.75	343	38.9	
	M40J	5.11	1.77	377	68.6	
	M50J	7.02	1.88	475	96.7	
	M60J	8.06	1.93	588	151.5	
Pitch-based carbon fibres	P25	5.9	1.9	159	22	
	P55	11.2	2	379	120	
	P75	14.6	2	517	185	
	P100	55	2.16	758	520	
	P120	61.3	2.17	827	640	
	K800	-	-	896	800	
	K1100	-	2.2	966	950	

Mesophase pitch-based carbon fibre was reported as a representative structural material with ultrahigh stiffness. Previous graphene fibre with disordered sheet-arrangement in transverse cross-section by simple wet-spinning shows low density and tremendously anisotropic single graphitic crystallites. While graphene fibre with optimal sheet-order along both axial and transverse direction shows improved density of 2.02 g cm^{-3} and enhanced three-dimensional crystalline size, inducing an obviously enhanced Young`s modulus.

Note:

[a] The crystalline sizes $La_{//}$ were measured by an empirical formula correlating the crystalline size with I_D/I_G collected from Raman spectrogram. The value was dramatically larger than that from X-ray crystallography.

Supplementary Table 3. Comparison of mechano-thermal properties of the graphene fibre and PAN/GO carbon fibre based on the concentric skeleton model with previously reported graphene fibre and PAN/GO carbon fibre without optimized sheet-order.

Category	Lable	Tensile strength (GPa)	Modulus (GPa)	Strain (%)	Thermal conductivity (W m^{-1} K^{-1})	Electrical conductivity (S m^{-1})	Reference
Concentric skeleton model	Graphene fibre	2.0	901	0.21	1660	1.21×10^{6}	This work
	PAN/GO carbon fibre	2.3	663	0.36	1254	0.77×10^{6}	
Random sheet-order	Graphene fibre	0.8	135	~0.61	1290	0.22×10^{6}	[2]
	PAN/GO carbon fibre	2.4	358	0.70	850	0.35×10^{6}	[4]

Supplementary Table 4. Specific modulus and specific thermal conductivity of graphene fibre and PAN/GO carbon fibre prepared by bidirectionally promoting assembly order, industrial metals, and conventional carbon fibres involving polyacrylonitrile (PAN)-based carbon fibre and Pitch-based carbon fibre.

Category	Lable	Specific modulus (MPa m^3/kg)	Specific thermal conductivity (mW m^2 /kg K)	Reference
Concentric fibres	Graphene fibre	433.2	826.9	This work
	PAN/GO carbon fibre	331.5	627.0	
Metal	Aluminium alloy	30.5	58.7	[14]
	Steel	25.2	2.1	
	Cu	12.3	44.6	
	Au	4.0	15.6	
	Mg	25.3	91.4	
	Ni	23.3	6.8	
	Ag	7.2	39.9	
PAN-based carbon fibres	T300	130.7	5.9	[4]
	T700	127. 8	5.2	
	T1000	163.3	17.8	
	M30J	169.9	18.4	
	M35J	196.0	22.2	
	M40J	213.0	38.8	
	M50J	252.7	51.4	
	M60J	304.7	78.5	
Pitch-based carbon fibres	P25	83.7	11.6	
	P55	189.5	60.0	
	P75	258.5	92.5	
	P100	350.9	240.7	
	P120	381.1	294.9	
	K1100	439.1	409.1	

Supplementary References

1 Xin, G. *et al.* Microfluidics-enabled orientation and microstructure control of macroscopic graphene fibres. *Nat. Nanotechnol.* **14**, 168-175 (2019).

2 Xin, G. *et al.* Highly thermally conductive and mechanically strong graphene fibres. *Science* **349**, 1083-1087 (2015).

3 Liu, Y. *et al*. Rapid roll-to-roll production of graphene film via intensive joule heating. *Carbon* **155,** 462-468 (2019).

4 Ming, X. *et al.* 2D-topology-seeded graphitization for highly thermally conductive carbon fibers. *Adv. Mater*. 34, 2201867 (2022).

5 Ma, W. *et al.* Systematic characterization of transport and thermoelectric properties of a macroscopic graphene fibre. *Nano Res.* **9**, 3536-3546 (2016).

6 Zhu, C. *et al*. A small-angle X-ray scattering study and molecular dynamics simulation of microvoid evolution during the tensile deformation of carbon fibres. *Carbon* **50**, 235-243 (2012).

7 Thunemann, A. F. *et al.* Microvoids in Polyacrylonitrile fibres: A small-angle X-ray scattering study. *Macromolecules* **33**, 1848-1852 (2000).

8 ANSYS Fluent Theory Guide, Release 21.2, ANSYS, Inc.

9 Menter, F. Zonal two equation kw turbulence models for aerodynamic flows. 23rd fluid dynamics, plasmadynamics, and lasers conference. 1993, 2906.

10 Mavriplis D. Revisiting the least-squares procedure for gradient reconstruction on unstructured meshes.16th AIAA computational fluid dynamics conference. 2003, 3986.

11 Poulin, P. *et al*. Superflexibility of graphene oxide. *P. Natl. Acad. Sci. USA* **113**, 11088-11093 (2016).

12 Plimpton, S. Fast parallel algorithms for short-range molecular dynamics. *J. Comput. Phys.* **117**, 1-19 (1995).

13 Li, P. *et al.* Highly crystalline graphene fibres with superior strength and conductivities by plasticization spinning. *Adv. Funct. Mater.* **30**, 2006584 (2020).

14 Metal, Plastic, and Ceramic Search Index (matweb.com)
