# Supplementary material for: Bidirectionally promoting assembly order for ultrastiff and highly thermally conductive graphene fibres
Source: Nat Commun. 2024 Jan 9;15:409. doi: 10.1038/s41467-024-44692-7 (PMC10776572; doi:10.1038/s41467-024-44692-7)
Supplement: Supplementary file 3 — Description of Additional Supplementary Files [file 41467_2024_44692_MOESM3_ESM.pdf]

Description of Additional Supplementary Files

Supplementary Movie 1. Observation of GO liquid crystals under rotating shear-flow.

Supplementary Movie 2. Tracking GO liquid crystals under unidirectional tubular shear-flow.

Supplementary Movie 3. Tracking GO liquid crystals under multiple shear-flow at rotating angular velocity of 100 (×2π/60) rad s^{-1}.

Supplementary Movie 4. Tracking GO liquid crystals under multiple shear-flow at rotating angular velocity of 500 (×2π/60) rad s^{-1}.

Supplementary Movie 5. 3D reconstruction of graphene aerogel fibres with concentric sheet-order.
